# Endogenous and xenobiotic metabolic stability of primary human hepatocytes in long-term 3D spheroid cultures revealed by a combination of targeted and untargeted metabolomics

**DOI:** 10.1096/fj.201601375R

**Published:** 2017-03-06

**Authors:** Sabine U. Vorrink, Shahid Ullah, Staffan Schmidt, Jatin Nandania, Vidya Velagapudi, Olof Beck, Magnus Ingelman-Sundberg, Volker M. Lauschke

**Affiliations:** * Section of Pharmacogenetics, Department of Physiology and Pharmacology, Karolinska Institutet, Stockholm, Sweden;; † Division of Clinical Pharmacology, Department of Laboratory Medicine, Karolinska Institutet, Stockholm, Sweden;; ‡ Metabolomics Unit, Institute for Molecular Medicine Finland (FIMM), University of Helsinki, Helsinki, Finland

**Keywords:** 3D cell culture, hepatic metabolism, drug metabolism, cytochrome P450 enzymes, mass spectrometry

## Abstract

Adverse reactions or lack of response to medications are important concerns for drug development programs. However, faithful predictions of drug metabolism and toxicity are difficult because animal models show only limited translatability to humans. Furthermore, current *in vitro* systems, such as hepatic cell lines or primary human hepatocyte (PHH) 2-dimensional (2D) monolayer cultures, can be used only for acute toxicity tests because of their immature phenotypes and inherent instability. Therefore, the migration to novel phenotypically stable models is of prime importance for the pharmaceutical industry. Novel 3-dimensional (3D) culture systems have been shown to accurately mimic *in vivo* hepatic phenotypes on transcriptomic and proteomic level, but information about their metabolic stability is lacking. Using a combination of targeted and untargeted high-resolution mass spectrometry, we found that PHHs in 3D spheroid cultures remained metabolically stable for multiple weeks, whereas metabolic patterns of PHHs from the same donors cultured as conventional 2D monolayers rapidly deteriorated. Furthermore, pharmacokinetic differences between donors were maintained in 3D spheroid cultures, enabling studies of interindividual variability in drug metabolism and toxicity. We conclude that the 3D spheroid system is metabolically stable and constitutes a suitable model for *in vitro* studies of long-term drug metabolism and pharmacokinetics.—Vorrink, S. U., Ullah, S., Schmid, S., Nandania, J., Velagapudi, V., Beck, O., Ingelman-Sundberg, M., Lauschke, V. M. Endogenous and xenobiotic metabolic stability of primary human hepatocytes in long-term 3D spheroid cultures revealed by a combination of targeted and untargeted metabolomics.

The liver is the principal organ responsible for xenobiotic metabolism and thus constitutes an important determinant of drug responses, with important implications for patients, health care providers, and the pharmaceutical industry. Drug-induced liver injury (DILI) is an important adverse drug reaction (ADR), with an estimated incidence rate of 13–19 cases per 100,000 individuals (1, 2), and constitutes the most common cause of acute liver failure in the Western world (3–5). Besides significantly contributing to patient morbidity and mortality, DILI is of significant economic concern for the pharmaceutical industry, because it is among the prime reasons for the attrition of drug development programs; 2% of all U.S. Food and Drug Administration–approved new medications between 1975 and 1999 displayed mandatory black-box warnings prompted by hepatic ADRs, causing reduced sales (6, 7). As a consequence of fewer approvals and an increasing number of withdrawals of products and sales restrictions, the average cost of new prescription medicines from development until approval were estimated at $2.6 billion U.S. (8).

Faithfully predicting drug metabolism and toxicity is thus of central importance for the pharmaceutical industry. Animal experiments are commonly required to obtain regulatory approval to progress into clinical stages. Yet, significant interspecies differences in structures, isoform compositions, expression, and catalytic activities of drug metabolizing enzymes result in poor concordance between animal and human toxicity (63 and 47% for human toxicity to nonrodents and rodents, respectively) (9, 10). Primary human hepatocytes (PHHs) are regarded as the current gold standard *in vitro* model to assess drug metabolism and toxicity (11). When cultured as conventional 2-dimensional (2D) monolayers, however, hepatocytes rapidly dedifferentiate (12–14), which significantly impairs their accuracy in predicting human *in vivo* drug metabolism (15). Consistent with this limited translational accuracy, a large-scale study of 7372 investigational drugs from 835 drug developers showed that the likelihood of approval of drug candidates entering clinical development was only 10.4%, with toxicity and unfavorable pharmacokinetics being responsible for most of the project closures (6, 16).

To overcome these obstacles, various hepatic 3-dimensional (3D) systems have been developed in which cultured hepatocytes remain viable and functional for prolonged times (17, 18). PHHs cultured in 3D cellular aggregates termed spheroids present a functionally and phenotypically stable, versatile system in which bile canaliculi are formed and hepatocytes retain their periportal and perivenous phenotypes (19, 20). Furthermore, this system has been demonstrated to have superior sensitivity for prediction of drug toxicity, when compared to other emerging hepatic cell culture systems, and to emulate hepatotoxicity of drugs with distinct toxicity mechanisms at therapeutically relevant concentrations (21). The utility of this platform as a predictive model for human drug response is showcased by fialuridine (FIAU), which caused the deaths of 5 of 15 participants in a clinical trial by inducing acute liver failure (22). Although FIAU toxicity was not detected in any preclinical model, including rat, mouse, dog, and cynomolgus monkey (23), the PHH spheroid system indicated that toxicity was already present at therapeutic exposure levels [spheroid EC_50_ = 100 nM; FIAU serum maximum concentration (*C*_max_) = 639 nM] (20, 24).

During preclinical stages of drug development, there is a need to predict human *in vivo* drug metabolism and pharmacokinetics, metabolite formation, time-dependent inhibition, and hepatic clearance, particularly of low-clearance compounds. If a cell system is to be successfully used as an experimental paradigm to derive accurate predictions about these parameters, physiologic and temporally stable phenotypes are necessary. Furthermore, an extensive experience and knowledge base is necessary that have to be generated by comprehensive characterization and relation to relevant comparator material. The PHH spheroid system has been comprehensively characterized by transcriptomic and proteomic analyses (20, 21). Yet, their metabolomic signatures have not been investigated. Liquid chromatography in combination with single or tandem mass spectrometry has been used for measuring *in vitro* cytochrome P450 (CYP) activity, as well as for targeted and untargeted metabolomics (25, 26). For quantification purposes, this technique requires the predefinition of analytes of interest through parameter optimization. For a more untargeted approach, high-resolution mass spectrometry (HR-MS) can be used, which can provide both precise quantification and untargeted data collection for metabolomic evaluation (27).

In this study, we systematically assessed metabolic signatures in PHH 3D spheroids over 3 wk of culture using Orbitrap HR-MS, which detects metabolites with highly divergent physical and chemical properties in a single analytical setup with maximum coverage (28). We benchmarked phenotypes and functionality of the spheroid system *vs.* fully mature hepatocytes and corresponding 2D cultures. We found that the endogenous and xenobiotic metabolic signatures of the system were stable overall and resembled metabolic patterns of freshly isolated cells, thus allowing comprehensive studies of drug-induced molecular effects on cellular metabolism and investigation of mechanisms of drug action (29–32). The results indicate that the 3D PHH spheroid system can be used for long-term analyses of drug metabolism and liver function and moreover is suitable for investigating *in vitro* metabolism of very low clearance drugs as well as for studying time-dependent inhibition of drug metabolism for relevant periods.

## MATERIALS AND METHODS

### Cell culture

Cryopreserved PHHs from 3 donors were commercially acquired from BioreclamationIVT (Brussels, Belgium) and were thawed in Cryopreserved Hepatocyte Recovery Medium (Thermo Fisher Scientific, Waltham, MA, USA). Demographic and medical information about the donors is provided in **Table 1**. Genotypes were determined with the CYP+ panel (PharmGenomics, Mainz, Germany). PHHs were seeded in 2D monolayer cultures into 12- or 96-well cell culture plates coated with 5 μg/cm^2^ rat tail collagen type I (Corning Inc., Corning, NY, USA) in culture medium (Williams’ medium E, supplemented with 2 mM l-glutamine, 100 U/ml penicillin, 100 µg/ml streptomycin, 10 µg/ml insulin, 5.5 µg/ml transferrin, 6.7 ng/ml sodium selenite, and 100 nM dexamethasone) with 10% fetal bovine serum. After attachment, the medium was replaced with serum-free culture medium and subsequently changed every 48–72 h. 3D spheroid cultures of cryopreserved hepatocytes from the same donors were seeded and maintained as has been described in Bell *et al*. (20).

**TABLE 1.** Demographic, serological, and medical information about the utilized donors

Donor	Sex	Age	Race	Cause of death	Medical history
1	M	58	Caucasian	CVA	Hypertension after 5 yr with medication; diabetes after 5 yr of noncompliance
2	F	48	Polynesian	Trauma	Diverticulitis with surgery
3	M	22	Caucasian	ICH/CVA	Hypertension at 18 yr; compliant, carcinoid tumor of appendix removed during appendectomy 4 yr ago; peritoneal dialysis: 2 kidney transplants 12 and 2 yr ago

CVA, cerebrovascular accident; ICH, intracerebral hemorrhage.

### Gene expression profiling

Gene expression analysis was performed by real-time quantitative PCR (qPCR) using TaqMan Universal PCR Master Mix (Thermo Fisher Scientific) and TaqMan probes (Supplemental Table S1). Data were collected with the ABI Prism 7500 sequence detection system (Thermo Fisher Scientific) and analyzed by using the ΔΔ*C_t_* method.

### Statistical analyses

Heteroscedastic 2-tailed Student’s *t* tests were used to determine *P* values, unless stated otherwise. Differences between metabolic activities of PHHs in 2- and 3D culture over time were compared by an extra sum-of-squares test using Prism 6 (GraphPad, La Jolla, CA, USA). The *r* values denote the Pearson product-moment coefficient.

### Sample preparation for targeted and untargeted metabolomics

We performed both an untargeted large-scale metabolomics approach and a targeted quantification of 56 endogenous metabolites (targeted metabolomics). When untargeted metabolomics is used, the identity of most of the metabolites cannot be unambiguously determined unless standards for the specific metabolites of interest are run. Thus, to complement this analysis, we ran standards for the metabolites of the CYP1A2, CYP3A4, CYP2C8, CYP2C9, and CYP2D6 probe substrates (Fig. 2*A*), which allows unambiguous identification and precise quantification of the metabolites in question. Standards for acetaminophen, dextrorphan, 4-hydroxytolbutamide, *N*-desethylamodiaquine, α-hydroxymidazolam, and α-hydroxymidazolam-d4 were purchased from Sigma-Aldrich (Round Rock, TX, USA). All solutions for all native substances were prepared in methanol, and working solution for internal standard was prepared in 0.3% formic acid.

Samples for untargeted metabolomics were prepared as follows: PHHs from 3 donors were seeded in a 2D monolayers (*n =* 3 biologic replicates per donor) and 3D spheroid cultures (*n =* 6 biologic replicates per donor) and were incubated with CYP probe substrates (10 µM midazolam, 15 µM dextromethorphan, 100 µM phenacetin, 10 µM amodiaquine, and 100 µM tolbutamide) for 4 h. Subsequently, supernatants were snap frozen, and the metabolites were quantified.

For targeted metabolomics of intracellular samples, cells were collected in fresh culture medium containing 25% acetonitrile, lysed by bullet blending, snap frozen and analyzed as indicated in Quantitative Targeted Metabolomics of Endogenous Metabolites.

### Untargeted metabolomics using HR-MS

The analyte separation on the HPLC system was performed on a Hypersil Gold C18 analytical column (100 × 2.1, 1.9 µm; Thermo Fisher Scientific). Total chromatographic run time was 14 min, with a gradient mode flow rate of 500 µl/min. The mobile phase consisted of 0.1% formic acid (v/v; solvent A) and acetonitrile with 0.1% formic acid (solvent B). The gradient profile was set as follows: starting with 2% B (hold time, 0.1 min) and continued with linear change to 45% B up to 12.5 min and 98% B up to 12.55 min. Continued 98% B up to 13.2 min and returned to the initial condition at 13.25 min, followed by equilibration until 14 min. The column oven temperature was 50°C, and the autosampler tray temperature was 10°C. The injection volume was 2 μl applied at mobile phase flow rate.

Untargeted mass spectrometric analyses were performed on an Orbitrap system (Q Exactive Plus) coupled to a Dionex Ultimate 3000 Ultra-High Performance Liquid Chromatography (UHPLC) system equipped with a binary pump, an autosampler, an online vacuum degasser, and a temperature-controlled column compartment. Instrumental operation, data acquisition and peak integration were performed with Chromeleon Xpress v. 3, Xcalibur v. 3.1, and Q Exactive, v2.6 (all systems from Thermo Fisher Scientific). The mass spectrometer was operated in positive electrospray ionization (ESI) mode with full scan. Source conditions for optimal sensitivity and selectivity were as follows: spray voltage, 3.0 kV; capillary temperature, 300°C; auxiliary gas heater temperature, 450°C; S-lens rangefinder level, 60; sheath gas, 50; and auxiliary gas, 18 (arbitrary units). The scan range was *m/z* 100–680 with resolution of 70,000 at *m/z* 200 (full width at half maximum). Two lock masses at *m/z* 214.0896 and 391.2842 were used.

### Quantification of CYP enzyme activity

From the untargeted metabolomic data set, acetaminophen, dextrorphan, 4-hydroxytolbutamide, *N*-desethylamodiaquine, and hydroxymidazolam were unambiguously identified by using internal standards with the exact monitored masses of *m/z* 152.0706, 258.1852, 287.1059, 328.1211, and 342.0803, respectively. Quantification was performed using extracted mass chromatograms from full-scan recordings with an *m/z* mass tolerance window of 5 ppm. Information about chromatographic separation and method validation is provided in the Supplemental Methods. Metabolomic analyses were performed with Compound Discovery software (Thermo Fisher Scientific).

### Quantitative targeted metabolomics of endogenous metabolites

Targeted metabolomic analyses were performed by liquid chromatography in combination with single or tandem mass spectrometry (33). In brief, 10 µl of labeled internal standard mixture was added to 100 μl of sample (cell lysate in Williams’ medium E buffer and 25% acetonitrile). Metabolites were extracted by adding 4 parts of the 100% acetonitrile+1% formic acid extraction solvent (1:4, sample: extraction solvent). The collected extracts were dispensed in Ostro 96-well plates (Waters Corp., Milford, CT, USA) and filtered by applying vacuum at a δ pressure of 300–400 mbar for 2.5 min on a robot vacuum station. Then, 5 μl of filtered sample extract was injected in an Acquity UPLC system coupled to a Xevo TQ-S triple quadrupole mass spectrometer that was operated in both positive and negative polarities with a polarity switching time of 20 ms for metabolite separation and quantification. Multiple reaction monitoring acquisition mode was selected for the quantification of metabolites. MassLynx 4.1 software was used for data acquisition, data handling, and instrument control. Data processing was performed with TargetLynx software (all equipment and software from Waters Corp.).

## RESULTS

### 3D culture of PHH significantly improves their phenotypes

**Figure 1.**
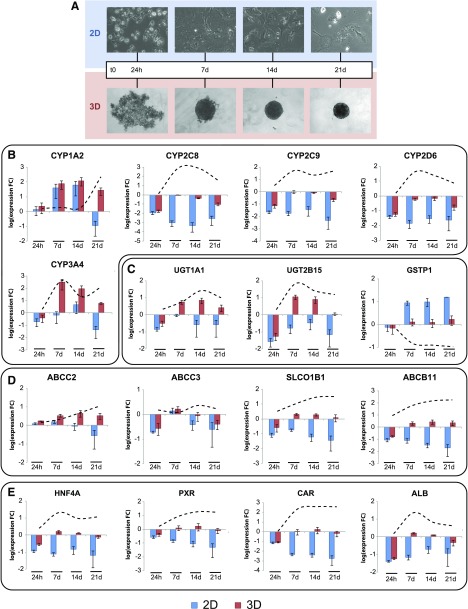
Hepatic expression signatures are preserved for multiple weeks in 3D PHH spheroid culture. *A*) Bright-field images depicting the temporal development of morphologic PHH phenotypes in 2D monolayer and 3D spheroid culture. Expression of phase I (*CYP1A2*, *CYP2C8*, *CYP2C9*, *CYP2D6*, *CYP3A4*; *B*) and phase II drug metabolizing enzymes (*UGT1A1*, *UGT2B15*, and *GSTP1*; *C*) drug and bile transporters (*ABCC2*, *ABCC3*, *SLCO1B1* and *ABCB11*; *D*), xenobiotic sensors (*CAR*, *PXR*; *E*), and hepatic markers (*ALB, HNF4A*; *E*) remain close to physiologic levels in 3D PHH culture, whereas expression was mostly lost in 2D culture of cells from the same donors (*n =* 3). The data are presented on semilog plots showing expression fold changes (FC) compared to freshly isolated cells. Dashed lines: the evolution of fold changes between 2- and 3D culture over time. Error bars = sem.

To comprehensively compare the effect of 2- and 3D culture methods on hepatic metabolic signatures, we first evaluated the temporal evolution of hepatic gene expression over the course of 3 wk by real-time qPCR analysis (**Fig. 1**). Expression levels of the important CYP enzymes *CYP2C8*, *CYP2C9*, and *CYP2D6* were rapidly and persistently downregulated in 2D culture (*P* < 0.01 for all genes and time points compared to freshly isolated cells), whereas their expression pivoted around physiologic levels in 3D culture (Fig. 1*B*). *CYP3A4* expression was similarly downregulated in 2D monolayer cultures, whereas expression in 3D spheroids was consistently upregulated. Expression of *CYP1A2*, which is regulated by the nuclear receptors aryl hydrocarbon receptor and constitutive androstane receptor (CAR), was strongly increased in 2- and 3D cultures (34, 35).

Similarly, genes encoding the phase II enzymes *UGT1A1* and *UGT2B15* were downregulated in 2D culture, whereas their expression did not differ significantly from isolated cells over the course of 3 wk in 3D culture (*P* > 0.1 for both genes and all time points). Expression of *GSTP1*, a marker for nonmature hepatocytes (36), rapidly increased in 2D monolayers, whereas it remained at physiologic levels in 3D spheroid culture (Fig. 1*C*).

Expression levels of *ABCB11*, encoding the bile salt export pump, and *SLCO1B1*, an import transporter for various endogenous and xenobiotic compounds (37), showed progressive decreases during dedifferentiation in 2D culture (*P* < 0.01 for both genes and at all time points), whereas they were not affected once spheroids were formed (*P* > 0.1 for 7, 14, and 21 d for both genes; Fig. 1*D*). In contrast, transcript levels of *ABCC2* and *ABCC3*, encoding the drug exporters multidrug resistance–associated protein (MRP)-2 and -3, respectively, did not significantly differ between the culture methods (*P* > 0.1 for both genes and all time points).

Pronounced changes in expression of the key hepatic transcription factor *HNF4A* and the nuclear receptors *NR1I3* (encoding CAR) and *NR1I2* [encoding pregnane X receptor (PXR)], which control the expression of many genes involved in absorption, distribution, metabolism, and excretion (ADME) of drugs (38, 39), differed significantly between culture methods (Fig. 1*E*). Whereas these genes were progressively downregulated in 2D monolayer culture as previously reported (14), their transcript levels were increased by 21-, 230- and 9-fold, respectively, in 3D culture and were indistinguishable from levels found in isolated cells. Thus, loss of key transcriptional regulators may provide an explanation for the observed differences in ADME gene expression between the culture systems.

### The functional stability of PHH is drastically extended in 3D culture

**Figure 2.**
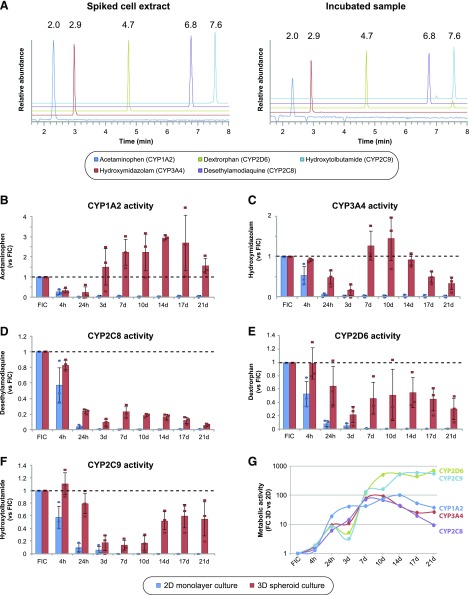
3D spheroid culture significantly improves the functional activity of major human CYP enzymes. *A*) Chromatograms of primary metabolites (acetaminophen, hydroxymidazolam, desethylamodiaquine, dextrorphan, and hydroxytolbutamide) of 5 CYP probe substrates in a calibrator (8 ng/ml) and in an incubated sample. *B–F*) Column plots showing the levels of the metabolic activities of CYP1A2 (*B*), CYP3A4 (*C*), CYP2C8 (*D*), CYP2D6 (*E*), and CYP2C9 (*F*) from 3 donors cultured in 2D monolayer and 3D spheroid culture. Dashed line: metabolite levels compared to freshly isolated cells (FICs). Error bars = sd. *G*) Line plot of fold changes between 2- and 3D cultures of the same donors (*n =* 3) demonstrate that metabolic activities are significantly elevated in 3D PHH spheroids.

Next, we compared the functional stability of xenobiotic metabolism in PHHs between 2- and 3D cultures. To this end, we used a cocktail of 5 noninteracting CYP probe substrates (midazolam, dextromethorphan, phenacetin, amodiaquine, and tolbutamide) and quantified their metabolites by liquid chromatography-quadripole extractive HR-MS (**Fig. 2**). The limits of quantification were <1 ng/ml for all analytes in blank cell extract. Figure 2*A* shows representative chromatograms of all metabolites in the blank cell extract and in an incubated sample.

Functional activities of CYP1A2, CYP2C8, CYP2C9, CYP2D6, and CYP3A4 exponentially declined and were reduced by 90% after 24 h and >95% after 7 d in 2D culture compared to freshly isolated cells (Fig. 2*B*–*F* and **Table 2**). In contrast, the metabolic activity of PHH in 3D culture was significantly higher for all 5 CYPs analyzed (*P* < 0.0001; extra sum-of-squares F-test). Although the functional capacities of hepatocytes were reduced during the initial aggregation stages (4 h, 24 h, and 3 d), they remained relatively stable once spheroids had formed (after 7 d), consistent with previous reports (20). Compared to freshly isolated cells, activities of CYP1A2 and CYP3A4 were increased to 230 ± 20 and 130 ± 20% (sem) after 7 d of 3D culture, whereas activities decreased to 23 ± 4, 46 ± 14, and 13 ± 5% for CYP2C8, CYP2D6, and CYP2C9, respectively. When quantitatively comparing the amount of metabolites formed per time between 2D monolayer and 3D spheroid culture, the functional activity in 3D culture was found to be elevated between 10- and 1000-fold across all CYP enzymes studied (Fig. 2*G*).

**TABLE 2.** Absolute levels of CYP activity of PHHs in 2D monolayer and 3D spheroid culture

Time	CYP1A2	CYP3A4	CYP2D6	CYP2C8	CYP2C9
3D spheroid culture					
** **FICs	9.4 ± 0.4	17.7 ± 1.2	4.3 ± 1.5	161.9 ± 17.7	7.9 ± 0.9
24 h	2.4 ± 1.7 (25.5)	8.7 ± 1.8 (49.2)	2.8 ± 1.5 (65.1)	37.1 ± 2 (22.9)	6.5 ± 1.3 (82.3)
3 d	14.6 ± 5.5 (155)	3.2 ± 1.6 (18.1)	0.68 ± 0.32 (15.8)	13.4 ± 2.7 (8.3)	1.5 ± 0.7 (19)
7 d	21.5 ± 4.5 (229)	21.8 ± 2.5 (123)	1.4 ± 0.4 (32.6)	36 ± 6.6 (22.2)	1.1 ± 0.5 (13.9)
10 d	21.5 ± 5.8 (229)	24.4 ± 4.1 (138)	1.3 ± 0.4 (30.2)	30.5 ± 4.9 (18.9)	1.2 ± 0.4 (15.2)
14 d	27.8 ± 1.5 (296)	16.4 ± 1.8 (92.7)	1.8 ± 0.4 (41.9)	26.2 ± 2.3 (16.2)	4 ± 0.5 (50.6)
17 d	26.2 ± 8.9 (279)	8.7 ± 1.5 (49.2)	1.6 ± 0.6 (37.2)	18.4 ± 2.1 (11.4)	5 ± 1.2 (63.3)
21 d	14.9 ± 2.8 (159)	5.6 ± 1.4 (31.6)	1 ± 0.4 (23.3)	9.8 ± 2.2 (6.1)	4.1 ± 1 (51.9)
2D monolayer culture					
FICs	14.3 ± 3	16.5 ± 2.5	5.5 ± 2.3	126.1 ± 23.6	9.7 ± 1
24 h	0.1 ± 0.09 (0.7)	0.69 ± 0.22 (4.2)	0.25 ± 0.13 (4.5)	4.25 ± 0.9 (3.4)	0.8 ± 0.27 (8.2)
3 d	0.36 ± 0.17 (2.5)	0.23 ± 0.14 (1.4)	0.1 ± 0.04 (1.8)	0.68 ± 0.07 (0.5)	0.41 ± 0.27 (4.2)
7 d	0.56 ± 0.23 (3.9)	0.28 ± 0.23 (1.7)	0.002 ± 0.002 (< 0.1)	0.36 ± 0.01 (0.3)	BDL
10 d	0.32 ± 0.13 (2.2)	0.27 ± 0.22 (1.6)	BDL	0.3 ± 0.03 (0.2)	BDL
14 d	0.31 ± 0.13 (2.2)	0.38 ± 0.31 (2.3)	BDL	0.46 ± 0.06 (0.4)	BDL
17 d	0.54 ± 0.24 (3.8)	0.34 ± 0.28 (2.1)	BDL	0.7 ± 0.12 (0.6)	BDL
21 d	0.44 ± 0.18 (3.1)	0.21 ± 0.18 (1.3)	0.002 ± 0.002 (<0.1)	0.69 ± 0.12 (0.5)	BDL
Other cell models					
Stem cell–derived HLCs	ND	ND	0.0003	ND	ND
HepG2	1.2	ND	0.67	ND	0.67
HepaRG	0.24–2.2	4	0.4	1	1.2–3.4

The probe substrates used to determine activities of CYP1A2, CYP3A4, CYP2D6, CYP2C8, and CYP2C9 were phenacetin, midazolam, dextromethorphan, amodiaquine, and tolbutamide, respectively. Activities are presented as rate of metabolite formation (pmol/min/10^6^ cells). The mean ± sem of 3 PHH donors is shown. Values for HepG2 and HepaRG cells as well as stem cell-derived HLCs were obtained from other publications (66–69). Where necessary, activity values provided per milligram protein were translated into per million cells, by using a conversion factor of 0.4 mg protein/10^6^ cells. Values in parentheses denote fraction of activity of freshly isolated cells (FICs). BDL, below detection limit; ND, not determined.

### Endogenous and xenobiotic metabolomic landscapes are maintained for at least 3 wk in 3D culture

**Figure 3.**
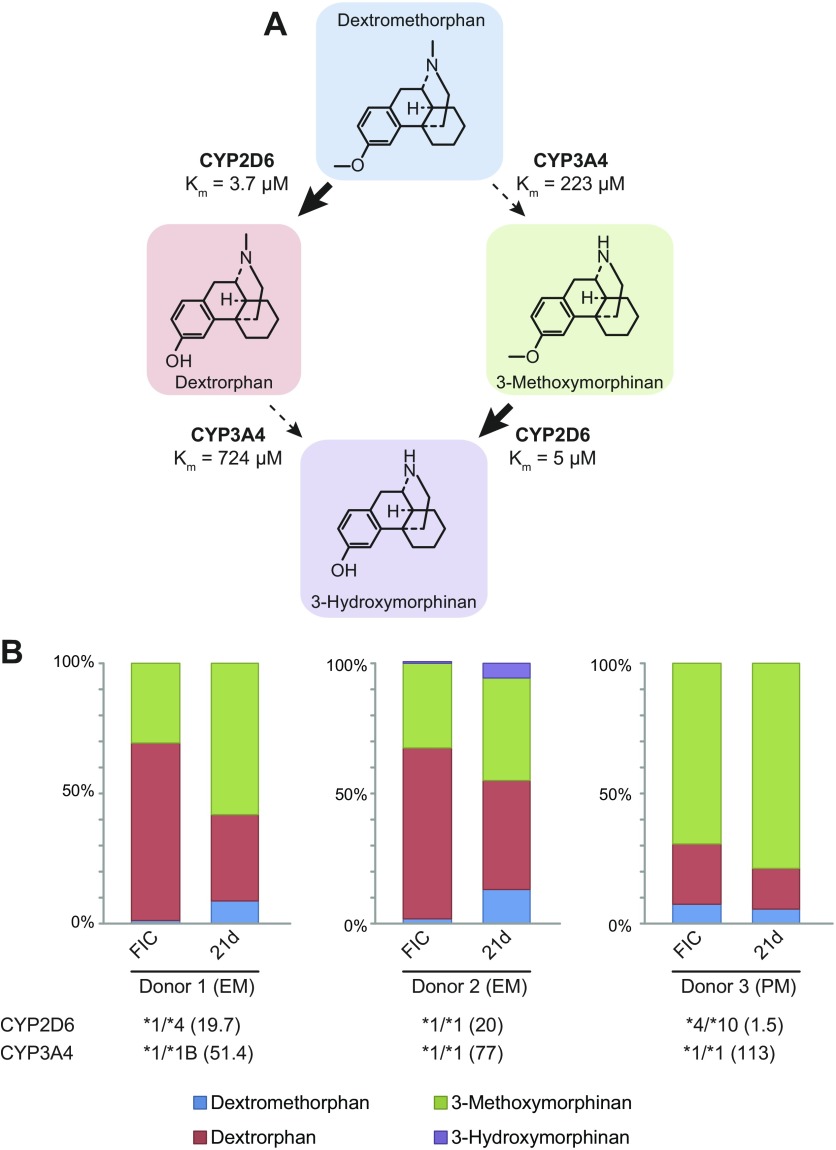
Interindividual differences in metabolic patterns are reflected in spheroid culture. *A*) Scheme visualizing different metabolic fates of dextromethorphan. *K*_m_ values were obtained from another publication (70). *B*) Metabolic profiles of dextromethorphan metabolism in PHH from 3 different donors in freshly isolated cells (FICs) and spheroids after 3 wk in culture. *CYP2D6* and *CYP3A4* genotypes were determined with a CYP+ panel. CYP2D6 and CYP3A4 were phenotyped directly after isolation by rate of formation of dextrorphan and 6β-hydroxytestosterone, respectively. Activity is presented in picomoles of produced metabolite per minute per million cells as provided by supplier. Donors were classified into extensive (EM) and poor (PM) metabolizers on the basis of phenotypic data.

We then quantitatively analyzed the metabolism of dextromethorphan to demonstrate the utility of this approach to assess the metabolic profile of candidate drugs. Dextromethorphan can be metabolized by CYP2D6 to dextrorphan and by CYP3A4 to 3-methoxymorphinan, which can be metabolized further to 3-hydroxymorphinan (**Fig. 3*A***). We analyzed 2 donors (CYP2D6*1/*1 and CYP2D6*1/*4) with indistinguishable metabolic activities that were phenotypically classified as extensive metabolizers (donors 1 and 2). Furthermore, we analyzed a donor who was phenotypically and genotypically categorized as a poor metabolizer (donor 3). This donor harbored one loss-of-function CYP2D6*4 allele and one *10 allele with reduced functionality and showed drastically reduced activity more than 10-fold lower than the other 2 donors (Fig. 3*B*). In both extensive metabolizers, most of the dextromethorphan was demethylated in freshly isolated cells and in spheroids after 3 wk in culture (Fig. 3*B*). Over culture time, the metabolic spectrum slightly tilted from CYP2D6-mediated *O*-demethylation to *N*-demethylation catalyzed by CYP3A4. Notably, dextromethorphan metabolism in the poor CYP2D6 metabolizer (donor 3) was strongly biased toward 3-methoxymorphinan in freshly isolated cells as well as after long-term spheroid culture in agreement with *in vivo* data (40). Combined, the presented data indicate that metabolic profiles are stable in 3D spheroid culture, and phenotypic differences observed *in vivo* can be successfully translated into an *in vitro* setting.

**Figure 4.**
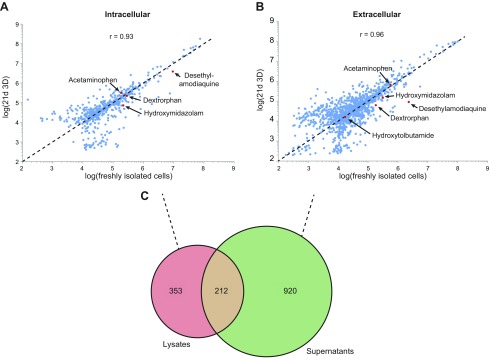
Intra- and extracellular metabolomes of 3D PHH spheroids remain stable over multiple weeks. *A, B*) Scatterplots of log intracellular (*A*) and extracellular (*B*) metabolite abundances at d 21 in 3D culture and in freshly isolated cells. For each metabolite, the average abundance of *n =* 6 biologic replicates is plotted. Red dots: probe substrate metabolites, unambiguously identified with internal standards. Dashed line: bisectrix corresponding to perfect correlation. The Pearson correlation coefficients indicate that metabolic profiles were stable over the course of 3 wk in culture. *C*) Venn diagram depicting the overlap between intracellular and extracellular compounds.

We then investigated the overall stability of PHH metabolomic signatures in 3D culture. Orbitrap HR-MS provided a comprehensive overview of intracellular metabolites, as well as the extracellular metabolic secretome of hepatocytes in spheroid culture (**Fig. 4*A*, *B***). First, we focused on metabolites of the 5 probe substrates. Few metabolites were found in extracellular and intracellular samples (acetaminophen, acetaminophen-sulfate, dextrorphan, hydroxymidazolam, and desethylamodiaquine), whereas hydroxytolbutamide and 3-hydroxymorphinan could be detected only extracellularly. When analyzing all identified endogenous compounds (*n*_extra_=1132 and *n*_intra_=565 distinct chemical entities), we found that relative concentrations were very similar with intra- and extracellular correlation coefficients of 0.93 and 0.96, respectively, between freshly isolated cells and spheroids after 3 wk in culture. Metabolites identified in intra- and extracellular compartments overlapped only to a limited extent (14.3%; 212/1485 identified metabolites), indicating that cell integrity is maintained (Fig. 4*C*).

### Quantitative analysis of endogenous metabolism in 3D PHH spheroid culture

**Figure 5.**
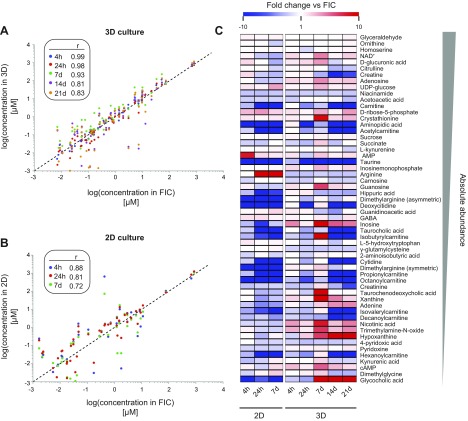
Concentrations of important endogenous metabolites remain stable in 3D PHH spheroid cultures. *A, B*) Log scatterplots of 56 endogenous hepatic metabolites in 3D spheroid (*A*) and 2D (*B*) culture. Concentrations in freshly isolated cells (FICs; *x* axis) are correlated with levels after 4 h, 24 h, 7 d, 14 d, and 21 d, and the Pearson correlation coefficient on log-transformed data is indicated. *C*) Heat map showing the temporal evolutions of metabolite levels of 56 basic physiologic compounds in 2D monolayer and 3D spheroid culture. Color coding depicts fold changes compared to freshly isolated cells. Metabolites are sorted in descending order of absolute concentration.

Because the metabolomic methodology that we used indicated only the relative overall stability of endogenous metabolism, we supplemented these findings with quantitative measurements of 56 intracellular metabolites with important endogenous functions (**Fig. 5**).

 In 2D monolayers levels of various metabolites were affected during early culture phases (Fig. 5*B*, *C*). After 4 h, a peak in AMP levels was detected exclusively in 2D but not in 3D cultures. In addition, we found that arginine levels increased progressively in 2D cultures and were upregulated more than 540-fold after 7 d, whereas they remained constant in 3D PHH spheroids throughout 21 d of culture. Furthermore, we observed major reductions in carnitine and carnitine-conjugate (isobutyryl-, isovaleryl-, propionyl-, octanoyl- and decanoylcarnitine) levels in 2D cultures and after prolonged culture periods (7 d) also in 3D cultures, indicating reduced mitochondrial import of fatty acids and thus decreased fatty acid β-oxidation.

In contrast, levels of most of the selected metabolites did not change over 3 wk in 3D spheroid cultures, reinforcing the relative data obtained by our comprehensive metabolomic approach (Fig. 5*A*, *C*). The concentration of aldose glyceraldehyde, an important molecule at the intersection between glycolysis and glycerol metabolism, was temporally invariant. Similarly, levels of arginine, ornithine, and citrulline remained stable over time, indicating the maintenance of functional urea cycle metabolism. Moreover, steady levels of glucuronic acid and UDP-glucose, which serve as a glycosidic substrate in endo- and xenobiotic conjugation reactions, in combination with maintained expression of responsible UDP-glucuronosyltransferase (UGT) enzymes (Fig. 1*C*), indicate stable phase II metabolism in 3D-cultured PHHs. Similarly, levels of NAD^+^ remained approximately constant during culture (2.6-fold change during 3 wk of 3D culture, which is within the range of physiologic fluctuation) (41).

Bile acid levels were rapidly and globally reduced in 2D culture (Supplemental Fig. S1). The rate-limiting enzyme in bile acid biosynthesis, CYP7A1, is regulated by HNF4A (42, 43) whose transcript levels were drastically reduced in 2D culture (Fig. 1*E*). In contrast, we observed stable bile acid levels in 3D culture; yet, with significant changes in bile composition. We observed a sharp drop in taurine levels, likely because the rate-limiting enzyme of taurine biosynthesis, cysteinesulfinic acid decarboxylase, is not expressed in human liver (44). Consequently, the taurine-conjugated bile acid taurocholic acid was rapidly lost, and cholic acid was instead conjugated to glycine, resulting in compensatory elevations in glycocholic acid concentrations (Supplemental Fig. S1).

Combined, these results suggest that key hepatic functionality, such as urea cycle and bile acid biosynthesis, were rapidly lost in 2D culture, whereas they were surprisingly stable in 3D spheroid culture on qualitative and quantitative levels for up to 3 wk in culture.

## DISCUSSION

Preclinical toxicity prediction of drug candidates and their metabolites is an integral part of all drug-development pipelines that encompasses *in silico*, *in vitro*, and *in vivo* models. Although animal testing constitutes the cornerstone of past and current safety assessments, there is growing recognition that pronounced species differences in hepatic metabolism impair the faithful translation of animal findings to humans (10). The usage of human cellular material has the potential to overcome these limitations. Yet, the confidence in conventional human *in vitro* models, such as hepatoma cell lines or primary hepatocytes in 2D culture, is also limited, as these simple systems do not accurately mimic human liver biology and function. To overcome these hurdles, a plethora of advanced 3D hepatic *in vitro* models have been developed that permit the maintenance of hepatic phenotypes for extended periods (18). However, to obtain regulatory approval substantial evidence for increased translational confidence has to be presented, which requires comprehensive characterization of these novel culture paradigms, as well as an extensive data and experience base (45).

In this study we extensively analyzed the transcriptional and metabolic profiles of PHHs in long-term 3D spheroid cultures and quantified the phenotypic improvements over conventional 2D monolayer cultures. In 3D cultures, expression levels of key regulators of hepatic gene expression profiles, such as *HNF4A*, and xenobiotic metabolism, including *CAR* and *PXR*, pivoted around levels found in freshly isolated cells, whereas they were downregulated 10- to 100-fold in 2D culture (Fig. 1*E*). Consequently, expression of genes with importance for hepatic functionality, including the HNF4A, PXR, and CAR targets *CYP2C8*, *CYP2C9*, *CYP3A4* and *UGT1A1* (46–49), were stable for at least 3 wk in the 3D model (Fig. 1*B*, *C*). In contrast, *GSTP1*, an indicator of undifferentiated hepatocytes (36) remained stable in 3D culture, whereas expression levels strongly increased in 2D (Fig. 1*C*).

When we correlated gene expression profiles to metabolic activities, we found that transcript levels were generally good predictors of metabolic capacities. Functional activities of CYP1A2, CYP2C8, CYP2C9, CYP2D6, and CYP3A4 were highly elevated in 3D compared to 2D culture (Fig. 2*B*–*F*), paralleling increases in expression of the corresponding gene transcripts. Furthermore, absolute functional activities were, in some cases, orders of magnitude higher than in systems based on hepatic cell lines or stem cell-derived hepatocyte-like cells (HLCs; Table 2). Notably, functional activities decreased during the initial stages of the spheroid aggregation process in agreement with reduced gene expression. Although expression levels of *CYP1A2* increased in 2D culture up to 100-fold, its functionality remained low (compare Figs. 1*B* and 2*B*), suggesting that post-transcriptional mechanisms are responsible for the uncoupling of activities between gene and gene product. On the basis of the functionality data, we estimated enzymatic half-lives of CYP1A2, CYP2C8, CYP3A4, CYP2D6, and CYP2C9 at 12, 12, 24, 36, and 75 h, respectively, which is in agreement with previously published *in vitro* and *in vivo* data (*t*_1/2_ for CYP1A2: 8–58 h, *t*_1/2_ for CYP2C8: 8–41 h, *t*_1/2_ for CYP3A4: 26 h, *t*_1/2_ for CYP2D6: 51 h and *t*_1/2_ for CYP2C9: 104 h) (50–52).

We then evaluated the metabolic flux in dextromethorphan metabolism in 2 donors who were phenotypically classified as extensive CYP2D6 metabolizers and 1 poor CYP2D6 metabolizer (Fig. 3). *In vivo*, metabolic extraction ratios of dextromethorphan:dextrorphan range between 0.001 and 0.1 in extensive metabolizers after 8 h (53). Similarly, we found that dextromethorphan was rapidly demethylated to dextrorphan in freshly isolated cells as well as in 3D PHH spheroids after 21 d in culture. After 4 h of incubation, only 2 and 11% remained methylated in extensive metabolizers, corresponding to 8 h extraction ratios of 0.0007 and 0.08 in freshly isolated and long-term cultured PHHs, respectively. In the poor metabolizer, dextromethorphan:dextrorphan ratios were higher (0.1 and 0.12 in freshly isolated cells and 21 d spheroids, respectively) than in extensive metabolizers approximating values reported *in vivo* (40, 53). These data provide proof of concept that metabolic profiles in PHH spheroids are stable over extended culture periods and that important interindividual differences that are common modulators of hepatic drug response can be translated into an *in vitro* setting (54). In the future, more comprehensive analyses with more donors will reveal whether this spheroid platform represents an *in vitro* paradigm capable of faithfully capturing the true pharmacokinetic patient diversity, which may enable simulation and prediction of the hepatic outcomes of clinical trials in a more cost-effective preclinical setting.

We then assessed metabolomic signatures measured by a new untargeted UHPLC–ESI–HR-MS quantification method, which has been shown to provide high-quality quantification results (55–57). Metabolic patterns and their responses to nutritional, viral, or xenobiotic challenges have been studied in primary rodent hepatocytes and human hepatic cell lines, such as HepG2, Huh7.5, and HepaRG, and such approaches have been used for mechanistic investigations and to predict DILI (58–62). Yet, without direct comparisons to physiologically relevant systems, such as mature fully differentiated PHHs, the translation of findings to humans is impaired. In this study, we addressed these shortcomings by performing time-course analyses in 3D PHH spheroid cultures, in which we related the metabolomic profiles after defined culture intervals to the metabolomic signatures of freshly isolated cells from the same donors, with unprecedented detectability. We found that the relative abundances of intracellular metabolites and the secreted metabolome were stable over time with correlation coefficients of *r* = 0.93 and 0.96, respectively (Fig. 4). Thus, 3D PHH spheroids accurately mimic endogenous metabolic profiles, even after multiple weeks in culture. To our knowledge, this study is the first to quantify the metabolic stability of any primary cell type in culture for extended lengths of time, which provides crucial information about their utility as long-term models for drug metabolism and hepatic functionality.

We complemented our metabolomic assessment with targeted and quantitative analyses of 56 important endogenous metabolites in 2- and 3D cultured PHHs. Early prominent changes in concentrations of the analyzed metabolites were detected in 2D monolayers early in the culture phases (Fig. 5). After 4 h of 2D culture, a peak in AMP levels was detected, suggestive of extensive metabolic remodeling, as AMP allosterically controls the activity of AMPK, a kinase that acts as an energetic sensor that can switch hepatic metabolism from anabolism to catabolism by phosphorylating target proteins, such as ACC1, CD36, and GS (63). Following these acute metabolic perturbations was a drastic increase in arginine levels that, combined with normal ornithine and citrulline concentrations, is indicative of ARG1 deficiency and urea cycle defects, as observed in fetal hepatocytes (64, 65). In contrast, metabolic signatures were stable overall in long-term 3D spheroid culture and important hepatic functions, such as urea cycle and bile acid biosynthesis, were maintained.

In summary, our data represent the first results that characterize the metabolomic stability of any primary human cell type in long-term culture. Endogenous metabolomic signatures, as well as the metabolic fluxes of xenobiotics, were found to be surprisingly stable for multiple weeks in 3D PHH spheroid cultures. Furthermore, the data indicate that, when all findings are related to freshly isolated cells from the same donors, overall transcriptional and metabolic profiles closely resemble physiologic patterns, thus incentivizing the use of this cell system as a physiologically relevant *in vitro* platform for studies of drug metabolism and pharmacokinetics, metabolite formation, time-dependent inhibition, or hepatic clearance.

## Abbreviations

2D: 2-dimensional
3D: 3 dimensional
ADME: absorption, distribution, metabolism, excretion
ADR: adverse drug reaction
CAR: constitutive androstane receptor
CYP: cytochrome P450
DILI: drug-induced liver injury
ESI: electrospray ionization
FIAU: fialuridine
HLC: hepatocyte-like cells
HR-MS: high resolution mass spectrometry
MRP: multidrug resistance–associated protein
NAD^+^: nicotinamide adenine dinucleotide
PHH: primary human hepatocyte
PXR: pregnane X receptor
qPCR: quantitative PCR
UDP: uridine diphosphate
UGT: UDP-glucuronosyltransferase
